# Selections

**Published:** 1892-05

**Authors:** 


### *SELECTIONS.*

---

OLIVE OIL FOR GALL-STONES.—In a recent clinical lecture Prof. Dujardin-Beaumetz (*Therap. Gazette*,) refers as follows to this method of treatment:

The facts are sufficiently numerous to warrant us in affirming that olive oil in large doses is one of the best means of combating the painful phenomena determined by the presence of biliary calculi. It arrests almost instantly the severe acute pains, and diminishes considerably the period during which the patients suffer dull pains, prostration and discomfort.

Failure constitutes the exception, and, what is strange, the large quantity of oil is well borne and the patients do not vomit. I say large quantity, for you must give in one dose 200 grammes (or nearly a tumblerful) of pure olive oil, and, in order to do away with its disagreeable taste, you can order the patient to rinse the mouth with brandy and water or to suck a little orange juice. In my own practice, to the olive oil I add bile, and with two hundred grammes of oil I combine twenty grammes of ox-gall. This mixture is slightly bitter, but it is well tolerated by the patient, and the results have been the same as with the oil, so that it is difficult for me to credit to the bile what really belongs to it in these cases. I was led to employ ox-gall by the researches of Prevost and Binet, who have shown that this substance is a powerful cholagogue.

We are still ignorant of the real mechanism of the therapeutic action of olive oil. Touâtre maintains that the oil always causes expulsion of the calculi. We know to-day the cause of this mistake. Touâtre confounded with gall-stones certain oily concretions resulting from the incomplete digestion of the ingested oil.

It is difficult to admit that the oil acts directly on the calculi, for we cannot conceive of its passing into the bile-ducts. Stewart maintained that the oil broke up into fatty acids and glycerin, and that the latter produced in the intestines reflex movements favoring the issue of the calculus. Others, and in particular

Rosenberg, considered the oil as a powerful cholagogue, and it is this cholagogue action which explains the favorable effects of the oil.

Lastly, we may suppose that the oil has a direct action on the orifice of the ductus communis choledochus and corresponding duodenal region, which tends to diminish the reflex spasm, which is the first cause of the colic.

For my part, I am ready to adopt the opinion of Willemin, who thinks that the large doses of oil act in several ways—first, as a cholagogue, then in diminishing the reflex action, and lastly in favoring the descent of the calculus into the intestine by their laxative properties.

However this may be, the number of favorable facts is to-day so considerable that, before having recourse to the injections of morphine, you ought always to make your gall-stone patients take a full dose 200 grammes of olive oil, with or without the addition of the ox-gall.—*Call and Clin. Rec.*

---

ANÆSTHESIA IN GYNÆCOLOGY.—The chapter on this subject in Pozzi's great work on gynæcology, as translated by Dr. Wells, and now being issued by Messrs. Wm. Wood & Co., of New York, is a most practical one for general as well as special surgery. We make the following extract:

Many German laparotomists use a mixture of chloroform and alcohol; the anæsthesia is said to be more uniform and vomiting less frequent. In France, chloroform reigns almost without a rival. Its purity should always be tested, especially if the anæsthesia is to be of long duration.

Under the same condition, and for particularly nervous and excitable patients, I have found it very advantageous to precede the administration of chloroform with a hypodermatic injection of morphine and atropine.

This should be given fifteen or twenty minutes before the chloroform. The resulting unconsciousness is more regular and of longer duration, although much less chloroform is required, and it also makes it easier to administer the anæsthetic with care. The process of mixed anæsthesia, which we owe to Dastre and

Morat, can scarcely be awarded too much praise in operations of any considerable length. It is designed to avoid the symptoms due to chloroform—laryngo-reflex syncope—especially secondary syncope. It prevents the initial excitement, diminishes nausea, limits the amount of chloroform used, and consequently lessens the chances of chloroform poison in operations of long duration. My learned friend, Prof. Dastre, has assured me that in his laboratory experiments, before he adopted this method, he lost one out of every four dogs anæsthetized. For the past ten years (1879–1889), he has used it upon hundreds of animals, and has not lost one. Safety and convenience are both gained by this process. It has been adopted by practical surgeons. Aubert head surgeon of the Antiquaille, in Lyons, uses it to the exclusion of all other methods, and testifies to its value in these words: "I know of nothing better nor more practicable. This method has the following advantages: 1. Safety. 2. More rapid induced unconsciousness. 3. Absolute calm on the part of the patient. 4. An easy awakening. 5. Very little malaise or vomiting as sequelæ. Many of my colleagues in Lyons, especially Profs. Gayet and Leon Tripier, have at my instigation used this method of anæsthesia. The number of cases experimented upon (1887) amounts to several thousands, with no resulting accident."

This mixture of morphine and chloroform was first used experimentally as an anæsthetic by Claude Bernard in 1864, and in surgery by Nusbaum. Further researches were carried on by Labbé and Guyon, Guilbert de Saint Briene, etc.—*Virginia Medical Monthly*.

---

ECTOPIC GESTATION.—Dr. Carter S. Cole describes, in the New York *Journal of Obstetrics and Gynecology*, how a case under his observation became converted from an extra-uterine into a normal pregnancy. Patient came under his notice in the second month of pregnancy. Had some irregularities of menstruation, morning nausea and dizziness, and colicky intermittent pains in abdomen. No changes in breasts. Vaginal examination disclosed uterus sharply anteflexed, and on the right side near the entrance of the tube an irregular mass, size of a small hen egg, distinct from the uterus and painless. Extra-uterine pregnancy at once suspected. Case was seen by Dr. Grandin in consultation, who confirmed the diagnosis and advised the use of galvanism, with everything in readiness for operation should occasion demand it. Patient at once put to bed, August 6th, and galvanic electricity applied, with the positive pole on abdomen and negative pole in vagina pressed well against the tumor and into the posterior *cul-de-sac*. *Seances* were repeated every two or three days, each lasting about fifteen minutes. On August 25th uterus was found enlarged, and examination revealed the disappearance of the tumor. The usual indications of normal pregnancy now supervened. The breasts became fuller, the morning sickness ceased and the abdomen began to enlarge.

Such cases—others similar to this have been reported—naturally raise the question as to the proper course to pursue during the prerupture stage of an ectopic pregnancy. Dr. Cole's conclusions are:

1. There is considerable—may we not say, incontrovertible—evidence that a strong probability of ectopic pregnancy can be determined in the prerupture stage.

2. The diagnosis in the prerupture stage can without operation seldom, if ever, be absolute.

3. We are not justified before rupture in an immediate laparotomy on a suspicion, however strong, unless there are peculiar and unusual conditions that will not warrant delay.

4. Electricity has proved itself, in intelligent hands, comparatively safe, often efficient, and is, in the prerupture stage, worthy of careful trial.

5. We should be prepared to do a laparotomy at any time if rupture supervenes, or if other considerations make operative interference imperative.

---

DR. GREWCOCK, in *London Medical Recorder*, says he found out quite accidentally a novel method of applying glycerine, which is equally efficacious with the clyster. If a piece of cotton wool alone, the size of a nut, is well saturated with glycerine and inserted as a suppository, in a short time a copious motion is produced.

THE INFLUENZA BACILLUS.—*Preliminary Communication on the Exciting Causes of Influenza.* By Dr. R. Pfeiffer, Chief of the Scientific Section.

The following results are based on the accurate examination of thirty-one cases of influenza, in six of which a necropsy was made. A complete report will be published as soon as possible.

1. In all the cases of influenza a bacillus of a definite species was found in the characteristic purulent bronchial secretion. In uncomplicated cases of influenza these tiny bacilli were found in absolutely pure cultures, and mostly in immense quantities.

2. These bacilli were found exclusively in cases of influenza. Very numerous control examinations proved their absence in ordinary bronchial catarrh, pneumonia, and pulmonary tuberculosis.

3. The presence of bacilli kept equal pace with the course of the disease; with the cessation of the purulent bronchial secretion the bacilli began to disappear.

4. I had already seen and photographed similar bacilli in the same enormous quantities two years ago, during the first epidemic of influenza, in preparations of the sputum of patients suffering from the disease.

5. The influenza bacilli appear as very tiny rodlets, of about the thickness of the bacilli of mouse-septicemia, but only half the length of these. One often sees three or four bacilli strung together in the form of a chain. They stain with some difficulty with the basic aniline dyes.

6. These bacilli can be obtained in pure cultures. On one and one-half per cent. sugar-agar the colonies appear as extremely small droplets, clear as water, often only recognizable with a lens. Their continued culture on this nutrient medium is attended with difficulties, and up to the present I have not succeeded in carrying it beyond the second generation.

7. Numerous inoculation experiments were made on apes, rabbits, guinea-pigs, rats, pigeons and mice. Only in apes and rabbits could positive results be obtained. The other species of animals showed themselves refractory to influenza.

8. In view of these results, I consider myself justified in pronouncing the bacilli just described to be the exciting cause of influenza.

9. It is very probable that infection is produced by sputum charged with the germs of the disease ; and the disinfection of the sputa of patients suffering from influenza is therefore urgently required as a prophylactic measure.—*Med. News.*

---

PIPERAZINE.—This is the name of a new uric acid solvent, which seems destined to replace the lithium salts in the treatment of urinary calculi. It appears as glassy lustrous crystals, perfectly soluble in water and practically tasteless. Internally, it is best given in weak aqueous solution, in doses of fifteen to twenty grains per day. In Schering's laboratory it was found that a sharp-edged fragment of a calculus, which consisted of successive layers of uric acid and was exceedingly hard, was completely dissolved in eight hours with a 1 per cent. Piperazine solution at blood heat (98 F.°). The sharp edges melted away and the surface of the calculus became slippery very shortly after first immersion.

A compact fragment of another calculus—composed of uric acid, calcium phosphate, and an indefinable organic substance—was dissolved in a 1 per cent. piperazine solution at blood heat, leaving only a light, porous skeleton, probably consisting of mucus.

The weight of a calculus, representing 0.7 grm. and consisting only of urate of ammonia, was reduced to 0.37 grm. by one day's immersion in a 1 per cent. piperazine solution.

Uric acid gravel was readily dissolved in 1 per cent. piperazine solution at blood heat, as was also crystallized uric acid.

Clinically, it appears that after two or three days use of a piperazine solution, a considerable evacuation of uric acid in the form of gravel is effected, due principally to the fact that the piperazine solution in washing the gravel from the kidneys makes it slippery, and thus the complete passage out is facilitated. Such prompt action often produces a feeling of relief and of security, but the use of the remedy—in the weak solution: 1 grm. of piperazine in a bottle of carbonated water as daily dose—

should be continued for several weeks more to insure complete eradication of the uric acid diathesis.

The new remedy promises to have an extensive and successful application in the uric acid diathesis, as well as against the diseases which are *sequelæ* thereto, such as gout, uric acid concretions, etc.—*New Remedies.*

---

RULES FOR THE ADMINISTRATION OF COCAINE.—Dr. Magitot, in the *Repertoire de Pharmacie*, formulates the following rules which should govern the employment of cocaine as an anæsthetic:

1. The dose of cocaine injected should be appropriate to the extent of the surface desired to render insensitive. It should not exceed in any case 1 to 1 3-4 grains. Each dose should be restricted in large surfaces.

2. Cocaine should never be employed in cases of heart disease, in chronic affections of the respiratory apparatus, or in nervous subjects; and this exclusion applies also to the other anæsthetics.

3. Cocaine should be injected into the interior, and not under the derm of the mucous membrane or of the skin. This is the intradermic method of Reclus, which should be substituted for the hypodermic method. By this means the introduction of a substance into the vein is avoided and the risk of accidents therefore minimized.

4. The injections should always be practiced upon the subject in a recumbent position, and he should only be raised when the operation is to be performed upon the head and mouth, and then only after anæsthesia is complete.

5. The cocaine should be absolutely pure, since, as pointed out by Laborde, its mixture with other alkaloids forms highly poisonous compounds.

6. Cocaine should be injected in divided doses, with a few minutes' interval.

7. Suspension of administration, or, as the author terms the method, "fractional injection," renders it possible to guard against the production of sudden symptoms of poisoning.—*Therapeutic Gazette.*

THE VALUE OF BICHLORIDE OF MERCURY IN THE TREATMENT OF URETHRITIS.—Brewer (*Internat. Jour. of Surg.*) reiterates his confidence in the efficacy of bichloride of mercury in the treatment of urethritis. His method was as follows: At the first visit the patient was instructed in the proper use of a syringe, and was given a large amount of a solution of bichloride of mercury, varying in strength from 1 : 16,000 to 1 : 50,000, according to the sensitiveness of his urethra and the stage of the disease. This he was instructed to use twice daily, by taking ten injections in the morning and ten at night, holding each one in the urethra one minute to imitate as nearly as possible the result of irrigation. The patient was seen three times a week. As soon as the discharge lost its purulent character, bichloride was suspended and a mild astringent was substituted, preferably bismuth suspended in water. In the fifty-five cases treated in this way, five were not benefited, after an average employment of the method for seven days. In the remaining fifty cases, the average length of time necessary to effect a change in the discharge from pus to thin watery secretion was a fraction over eight days. The discharge entirely disappeared on an average of twenty-one days. Epididymitis occurred in three of these cases and posterior urethritis was developed in two. The reporter very fairly states that these statistics are of little value from a scientific point of view, since the cessation of the discharge can by no means be considered as an index that the disease has been cured. He offers his conclusions not so much on the basis of these cases as upon a very large personal experience, and states positively that the judicious use of bichloride of mercury in cases of acute gonorrhœal urethritis is attended with better results in subduing the painful and disagreeable features of the disease than in any other agent. The recovery is more rapid and permanent, and the frequency of inflammatory complications is very greatly reduced.—*Therapeutic Gazette.*

THE EYE.—Never prescribe for an inflamed eye without doing three things, viz.:

1. Without examining for a foreign body imbedded in the cornea, or lodged beneath the lids.

2. Without seeing if cornea or iris is implicated.

3. Without determining the presence or absence of tension of globe.

Never use violence in opening the eye, if there be much swelling or spasm, because if there be a deep ulcer of the cornea present, perforation may take place.

Never apply lead lotion (Goulard water) should there be the slightest abrasion of the corneal epithelium. (Solid particles of oxide or carbonate of lead become deposited and form permanent opacities.)

Never trust the nurse with verbal instructions for washing out the baby's eyes in infantile ophthalmia. Do it yourself.

Never forget that wounds of the ciliary region are most dangerous, and if they involve the lens, or if they are attended with loss of vitreous, they need excision of the eye.

Never put atropine into an eye:

1. Without testing tension.

2. Without examining for locomotor ataxia (for ataxial cases walk by sight).

3. Without due care as to strength in old people.

(N. B. Beware of atropine, ergot, colchicum in old people.) —*Medical Brief.*

---

TREATMENT OF MIGRAINE.—Dr. C. W. Suckling, Birmingham, England, recommends:

℞. Ext. cannabis indicæ............ gr. $\frac{1}{6}$

Zinci phosphidi.................. gr. $\frac{1}{10}$

Acidi arseniosi.................. gr. $\frac{1}{30}$

Rest in bed in a dark room may be necessary, and relief may be secured by a draught every hour consisting of ten grains each of antipyrin and ammonium bromide with twenty minims of sal volatile.—*Birmingham Medical Review.*

A DELICATE TEST FOR ALBUMIN IN THE URINE.—Spiegler (*Wiener klinische Wochenschift*, January 14, 1892) suggests the following formula for the discovery of albumin in the urine as being the most delicate test we possess:

| | | |
|---|---|---|
| ℞. | Hydrarg. chlor. corrosiv................ | 8 |
| | Acid. tartar. .................................. | 4 |
| | Aq. dest.........................................| 200 |
| | Sacch. alb......................................| 20 |

It is used as follows: The test tube is filled one-third with the reagent. The urine is filtered and made strongly acid with acetic acid. It is then allowed to flow down the side of the tube, drop by drop, until it lies in a layer over the reagent. If albumin is present, a sharp white ring is seen lying between the two layers of fluid. If it is necessary to test heavy diabetic urine, more sugar may be added to the reagent, in order to raise its specific gravity. In the absence of mucus it is necessary to decompose any carbonate that may be present, in order that it may not form a precipitate with the sublimate. But the precipitate may be recognized by the fact that when shaking the liquid the apparently caseous precipitate will disappear and the fluid become clear.—*Univ. Med. Mag.*

---

INJECTIONS FOR CHRONIC CYSTITIS.—Ultzmann recommends the following prescriptions in the treatment of this troublesome condition:

| | |
|---|---|
| Crystallized carbolic acid.................. | gr. xv. |
| Distilled water.................................. | ℥iiiss. |

Dissolve, and mix with equal parts of hot water at the moment that the liquid is to be injected.

Or the following:

| | |
|---|---|
| Boric acid.......................................... | ℥ss. |
| Glycerin.............................................. | ℥i. |
| Distilled water.................................. | ℥x. |

Make a solution, and mix with equal parts of warm water at the moment of employment.—*Buff. Med. and Surg. Journal.*

ETHER AND THE KIDNEYS.—Dr. Feutin, of Borne, thus concludes, from experiments on animals and upon 150 cases wherein ether was given, the time noted, and the amount consumed:

1. That ether has no perceptible effect upon the healthy kidneys of animals—who, moreover, are more susceptible than mankind to its influence.

2. That it is not dangerous in persons whose kidneys are slightly diseased.

3. That subsequent disturbances of circulation in the kidney, when met with, are very transitory and rapidly disappear.

4. That the presence of abnormal urinary ingredients is not a positive counter-indication for the use of ether.—*Journal American Medical Association.*

---

RHEUMATISM.—In the treatment of this malady in many of its varieties, I have found no prescription equal to the following:

| | | |
|---|---|---|
| ℞. | Bicarbonate potassium, Salicylic acid, Iodide potassium, } .........āā | 2 drachms. |
| | Tinct. colchicum seed............. | 3 drachms. |
| | Syrup orange peel................. | 3 ounces. |
| | Water.................................... | 5 ounces. |

M. Shake well. Sig.: A tablespoonful every two or three hours until necessary to diminish the dose and its frequency.—*J. B. Johnson, in Southern Clinic.*

---

DELIRIUM TREMENS.—The following is highly spoken of in delirium tremens:

| | | |
|---|---|---|
| ℞. | Chloral hydrat.,............... | ʒjss. |
| | Potass. bromid.,........... ... | ʒij. |
| | Spirit. ether comp., ....... | fl. ʒij. |
| | Tinct. valerianæ,............ | fl. ʒiij. |
| | Aquæ,....................... | fl. ℥vj. |

One teaspoonful every two, three or four hours until the patient becomes quiet.—*The Prescription.*

